# 1593. Switch to DOVATO in Patients Suppressed on Biktarvy (The SOUND Study), week 48 interim analysis

**DOI:** 10.1093/ofid/ofad500.1428

**Published:** 2023-11-27

**Authors:** Jihad Slim, James Fallon, Wassim Abouzeid, Helen Fadoju-Oloyede, Amtus Shafiq, Ala Muhanna

**Affiliations:** Saint Michael’s Medical Center, Newark, NJ, USA, Newark, New Jersey; Saint Michael's Medical Center, Newark, New Jersey; Saint Michael's Medical Center, Newark, New Jersey; Saint Michael's Medical Center, Newark, New Jersey; Viiv Healthcare, RTP, North Carolina; Saint Michael's Medical Center, Newark, New Jersey

## Abstract

**Background:**

Dolutegravir/lamivudine(DTG/3TC) is a complete regimen, approved by the FDA for the treatment of HIV-1 infection in treatment-naïve or virologically suppressed patients with no prior virologic failure or resistance to DTG/3TC. The SOUND study evaluated virologically suppressed (< 50 copies/ml for >6 months) patients on bictegravir/emtricitabine/tenofovir alafenamide(B/F/TAF) for >24 weeks, with an unknown HIV resistance history who were switched to DTG/3TC.

**Methods:**

SOUND recruited patients from urban clinics in the Northeastern U.S. serving predominately African American or Latinx communities is a prospective open-label, single-center, pilot study with the primary endpoint % of patients with HIV-1 VL ≥ 50 copies/mL at week 48; Secondary endpoints at weeks 48 and 96 include percentage of patients with HIV-1 VL < 50 copies/mL, incidence and severity of adverse events (AEs) and laboratory abnormalities, retrospective pro-viral DNA testing on banked samples at baseline to compare virologic outcome (with or without M184V/I), number and type of resistance-associated mutations in virologic failure.

**Results:**

We enrolled 40 patients suppressed on B/F/TAF, 18 (45%) female, 23 (57.5%) Black, mean time on B/F/TAF 2.75 years (range: 1 – 3.6), mean time since HIV diagnosis 16.4 years (range: 1.2 – 36), mean number of prior antiretroviral regimen before entering the study 5 (range 1 – 14), baseline CD4: 681 cells/ml (range:372-1044). Nadir CD4 mean: 364(range:13-734). At week 48, 37 (92.5%) patients were virologically suppressed. Three patients withdrew informed consent and were virologically suppressed at the time of discontinuation. Five patients experienced serious adverse events none were related to study medications, and no one stopped DTG/3TC due to AE.

**Conclusion:**

This pilot study supports the switch to DTG/3TC in patients virologically suppressed on B/F/TAF, with unknown resistance history.

**Disclosures:**

**Jihad Slim, MD, FACP**, ViiV Healthcare: Advisor/Consultant|ViiV Healthcare: Grant/Research Support

